# The effect of omega-3 fatty acids on clinical and paraclinical features of intractable epileptic patients: a triple blind randomized clinical trial

**DOI:** 10.1186/s40169-019-0220-2

**Published:** 2019-01-16

**Authors:** Shaghayegh Omrani, Mohammad Taheri, Mir Davood Omrani, Shahram Arsang-Jang, Soudeh Ghafouri-Fard

**Affiliations:** 1 Department of Neurology, Hamadan University of Medical Sciences, Hamadan, Iran; 2 Student Research Committee, Shahid Beheshti University of Medical Sciences, Tehran, Iran; 3 Urogenital Stem Cell Research Center, Shahid Beheshti University of Medical Sciences, Tehran, Iran; 4 Clinical Research Development Center (CRDU), Qom University of Medical Sciences, Qom, Iran; 5 Department of Medical Genetics, Shahid Beheshti University of Medical Sciences, Tehran, Iran

**Keywords:** Omega 3, Refractory seizure, Clinical trial

## Abstract

Long chain omega-3 fatty acids (omega-3 FAs) supplements have been shown to exert beneficial effects in patients with epilepsy through elevation of seizure thresholds and dampening of inflammatory responses. In this triple blind randomized, placebo-controlled parallel group trial of omega-3 FA supplementation, 180 mg eicosapentaenoic acid (EPA) and 120 mg docosahexaenoic acid (DHA) as well as placebo capsules were administered twice a day in 50 patients with refractory seizure during a 16-week period respectively. Seizure frequency and duration were reduced after completion of the treatment in the supplement group. The supplementation resulted in a significant decrease in TNF-α and IL-6 concentrations. Further studies are needed to compare different omega-3 FA compositions and determine the most effective dose and treatment duration as well as the long term benefits of this supplementation.

## Introduction

Epilepsy as one of the most common neurologic disorders has led to significant morbidity and mortality [1]. Approximately 40% of epileptic patients are unresponsive to pharmacological management and are characterized as having refractory seizures. The International League Against Epilepsy has defined intractable epilepsy as the lack of success in seizure control by sufficient trials of 2 tolerated and properly selected and used antiepileptic drug (AED) programs [2]. Non-pharmacological treatment modalities including administration of omega-3 fatty acids (FAs) have been suggested to be effective in control of seizure frequency in refractory epileptic patients [3]. These kinds of FAs as principal elements of neuronal membranes participate in synaptic plasticity, neuroimmune-modulation and neuron preservation [4]. Docosahexaenoic acid (DHA) as the main omega 3 polyunsaturated FA of the brain tissue exerts anti-epileptic effects in animal models as demonstrated by increase in the latency to onset of pentylenetetrazol seizures in addition to elevated focal and generalized cortical seizure thresholds [5]. Other omega 3 polyunsaturated FAs, such as α-linolenic acid (ALA) or Eicosapentaenoic acid (EPA) make up a minor proportion of total brain fatty acids [6]. The beneficial effects of omega-3 FA supplementation on reduction of seizure frequency and elevation of seizure threshold have been documented in human clinical trials as well [7].

The epileptogenesis process has been linked with the over-production of pro-inflammatory cytokines such as tumor necrosis factor (TNF)-α and interleukin (IL)-6 as demonstrated by increased susceptibility to seizures in inflammatory disorders such as colitis, pneumonia and rheumatoid arthritis [8]. In addition, several studies have demonstrated the malfunction of the blood–brain barrier following the administration of pro-inflammatory cytokines IL-6 and TNF-α which might be associated with predisposition to seizure [9]. IL-6 and TNF-α have been the most studied cytokines in relation with epileptogenesis [10]. Animal studies have shown neurotoxic and pro-convulsive effect of both IL-6 and TNF-α in the brain [10]. The levels of TNF-α and IL-6 have been increased in glial cells after epileptic seizures [9]. Vagus nerve stimulation has been shown to alter IL-6 plasma levels in patients with refractory epilepsy [11]. Cytokine levels have been suggested as predictors of the beginning of spontaneous seizures as well as appropriate biomarkers for revealing brain damage in high-risk epileptic patients [9]. Moreover, IL-6 and TNF-α have been the most studied cytokines in association with omega-3 FAs. For instance, Battle et al. have shown association between consumption of omega-3 FAs and serum levels of TNF-α in chronic obstructive pulmonary disorder [12]. Barkhordari et al. have reported a significant reduction in IL-6 levels following elevation of serum DHA in rat [13]. Finally, Kalogeropoulos et al. have demonstrated a significant positive correlation between plasma omega-6/omega-3 ratio and levels of IL-6, TNF-α in healthy subjects [14].

In the present study, we evaluated the effects of omega-3 FA supplementation on clinical and paraclinical features of refractory epilepsy including the levels of pro-inflammatory cytokines in a triple blind clinical trial.

## Materials and methods

### Patients

This was a 16-week triple-blind, placebo-controlled, parallel group trial of omega-3 FA supplementation in 50 patients with refractory epilepsy. Patients aged between 18 and 55 years were enrolled in the study if their 16-week retrospective seizure counts were clearly recorded and showed occurrence of generalized epileptic seizures despite of treatment with maximum tolerable doses of three AED. Patients with hepatic diseases (more than 2 times elevation of liver enzymes), renal diseases, active infections, diabetes, hypertension, dementia or mental retardation as well as pregnant or breast-feeding women were excluded from the study. Blood samples were taken before and at the end of the 16-week triple-blind treatment phase to assess TNF-α and IL-6 concentrations. Seizure frequencies and duration were recorded during the study. Seizure frequency was assessed by documentation of the number of epileptic attacks per month using a memoir given to the patients or their parents (if the patients aged < 18) to record the number of seizures each day.

The block randomization method was used to randomize patients into two groups with identical sample sizes. The block size was determined to be six (three blocks marked with A and three with B). Blocks were randomly selected to assign patients into the groups. The staff that was responsible for giving supplement/placebo was unaware of coding system. The study was designed as a triple blind study. Patients, the neurologists who assessed the patients and the statistician did not know the kind of administered supplement. Only a coordinator was aware of the condition. The Independent Data Monitoring Committee strategy was applied.

Omega-3 fatty acids capsules containing 120 mg of DHA and 180 mg of EPA plus vitamin E (Zahravi Pharmaceutical Company, Tehran, Iran) and matching placebo capsules were administered twice a day in each study group respectively. The daily dose and duration of the trial were chosen based on the previously reported optimal doses and durations in patients with refractory epilepsy [7, 15] as well as the availability of the standardized formulation. Patients were followed by trained staff to confirm uptake of capsules. In both groups, the AED treatments were continued. The study protocol was approved by the Ethics Committee of Hamadan University of Medical Sciences (IRCT201604179014N96). Written consent was obtained from all participants. Patients’ characteristics are summarized in Table 1.

**Table 1:** The characteristics of patients in each study group

Patients characteristics	Placebo	Supplement
Number of subject	25	25
Male: female	13:12	15:10
Age (year)	18–55	18–55
Epilepsy syndrome		
Grandmal seizures	25	25
Antiepileptic drugs (AED)		
Carbamazepine	13	9
Valproate	16	15
Dilantin	6	9
Lamotrigen	5	7
Levebel	5	10
Clobazam	1	0
Clonazepam	8	0
Primidone/phenobarbital	7	10

### Cytokine detection

Three milliliters of peripheral venous blood sample obtained from participants were used for detection of plasma concentrations of TNF-α and IL-6 levels at the beginning of the study as well as the end of treatment phase. Serum cytokine levels were assessed by commercially available ELISA kits (BOSTER BIOLOGICAL TECHNOLOGY, LTD, Pleasanton, CA, United States) based on the manufacturer’s protocol on Infinite M200 (Tecan, Männedorf, Switzerland) microplate reader.

### Electroencephalogram (EEG) patterns

EEG patterns were recorded at baseline and after the supplementation period in each study group. The background activity was classified into three groups based on the formerly described criteria [16] with some modifications consistent with the new American Clinical Neurophysiology Society guidelines [17]: (1) normal/mild pattern defined by constant background activity with little abnormal activity such as mild asymmetries, mild voltage depression, poorly defined sleep–wake cycling, (2) moderate pattern described by intermittent activity with interburst interim ≤ 10 s, no distinct sleep–wake cycling or obvious asymmetry or asynchrony, (3) severe pattern demonstrated by intermittent activity with interburst interim 10–60 s, severe diminution of background activity, no sleep–wake cycles.

### Primary outcome assessment

Seizure frequencies and durations were assessed each month post randomization and compared with 16 weeks immediately prior to randomization.

### Secondary outcome assessment

Cytokine levels and EEG pattern were evaluated at the beginning of the study and after completion of treatment period.

### Statistical analysis

The quantitative data are presented as the mean (± standard error) and median (quartiles 25%, quartile 75%). The independent T test and Chi-Square tests were applied to evaluate the baselines differences between the study groups. The robust Analysis Of Covariance (ANCOVA) test was used to examine the effects of intervention on clinical and paraclinical parameters. The WRS2 package in the R 3.4.2 program was used to compute robust ANCOVA for 2 independent groups with adjusting the covariates. No parametric assumption was made about the form of data. The bootstrap method was used for determination of confidence intervals and *P* values. The analyses were adjusted for sex and age group interaction using baseline measurements as covariates. Pearson’s Chi-squared test was applied for evaluation of differences in EEG grades between two study groups after treatment period.

## Results

The study included 50 patients with refractory epilepsy randomly divided to two groups (Supplement and Placebo). Baseline values are shown in Table 2. Twenty percent of patients in each study group had a baseline severe EEG pattern demonstrated by intermittent activity with interburst interim 10–60 s, severe diminution of background activity and no sleep–wake cycles. There was no report of side effects in any of study participants. All patients continued the study period. There were no statistical differences between two groups prior treatment in any of the assessed parameters (*P *> 0.05). Moreover, there was no significant difference in patients’ sex (χ^2^ = 0.325, *P *= 0.569) and EEG patterns (χ^2^ = 2.05, *P *= 0.638) between treatment and placebo groups.

**Table 2:** Baseline measurements in the study groups

Parameters	Study groups	Mean ± SD	Median [IQR]	*P* value
Age	Supplement	34.08 ± 10.6	32 [27.5, 41.5]	0.676
	Placebo	32.88 ± 10.7	32 [24.5, 40.5]	
Seizure frequency in recent 4 months	Supplement	6.8 ± 4.3	5 [4, 10]	0.83
	Placebo	8.36 ± 7.3	6 [3.5, 11]	
TNF-α levels	Supplement	24.98 ± 38.36	13.02 [3.67, 26.4]	0.258
	Placebo	43.27 ± 46.7	22.4 [8.64, 68.25]	
IL-6 levels	Supplement	4.37 ± 3.02	4.03 [2, 6.09]	0.684
	Placebo	6.76 ± 11.58	4.37 [1.56, 6.43]	
Epilepsy duration in recent 4 months (min)	Supplement	13.88 ± 7.45	10 [10, 20]	0.226
	Placebo	17.04 ± 9.07	10 [10, 30]	

All parameters were changed in both groups after the 16-week period of study except for seizure frequency in placebo group which was unchanged. A significantly higher fraction of patients receiving supplements (9/25) compared with those receiving placebo (5/25) had no seizures during the study period. Based on the results of ANCOVA test and considering the baseline values of all parameters, TNF-α and IL-6 levels were significantly lower in supplement group compared with placebo group. In addition, frequency and duration of epilepsy were significantly lower in supplement group compared with placebo group. Median of TNF-α levels has been decreased 38% in supplement group while increased 77% in placebo group. Table 3 shows the results of effects of supplement treatment on assessed parameters.

**Table 3:** ANCOVA results for comparison of two groups with adjustment of age, gender and baseline measurements

Parameters	Study groups	Mean ± standard error of mean	Median of percent change (%)	Mean difference	*P* value	95% confidence interval for differences	Effect size
Seizure frequency	Supplement	4.72 ± 1.6	− 80	− 6.91	0.014	[− 11.26, − 2.79]	0.442
	Placebo	11.64 ± 1.63	0				
TNF-α	Supplement	13.43 ± 4.4	− 38.27	− 43.4	0.002	[− 61.67, − 25.6]	0.703
	Placebo	74.14 ± 13.3	77.33				
IL-6	Supplement	2.28 ± 0.37	− 41.23	− 5.21	0.004	[− 7.5, − 3.2]	0.725
	Placebo	7.53 ± 1.02	46.41				
Epilepsy duration in study period (min)	Supplement	6.64 ± 1.39	− 50	− 6.061	0.009	[− 10.12, − 1.9]	0.408
	Placebo	14.36 ± 2.18	0				

All results are based on Bootstrapping Method; Effect size conventions; f = 0.1: small, f = 0.25: medium, f = 0.4: large

Evaluation of EEG patterns showed no change in 44%, one-point improvement in 40%, two points improvement in 4% (changing from severe diminution of background activity to mild voltage depression) and one-point worsening in 12% of patients (change in interburst interim from ≤ 10 to 10–60 s) supplemented with omega-3 FAs. In placebo group, 54% of patients had no change in EEG grade while 20% and 16% of patients had one and two points worsening in EEG grades respectively (demonstrated by change in interburst interim or background activity). Pearson’s Chi-squared test showed significant difference in EEG grades between tow study groups (Pearson’s Chi-squared = 9.43, Monte Carlo P value = 0.034).

## Discussion

In the present triple blind clinical trial, we demonstrated beneficial effects of omega-3 FA administration in reduction of seizure frequency and duration in patients with refractory seizures. FAs have been previously shown to alter ion channel activities and modify electrical signal transduction [18]. Of note, in the current study the levels of TNF-α and IL-6 were significantly decreased in patients received omega-3 FA supplements compared with placebo group. Previous studies have demonstrated that omega-3 FAs diminish inflammation through inhibition of the formation of pro-inflammatory eicosanoids, suppression of the activity of nuclear transcription factors, such as NF kappa B, and decreasing the release of pro-inflammatory enzymes and cytokines, including TNF-α [19]. Besides, the association between dietary intake of omega-3 FAs and serum levels of TNF-α has been established in chronic obstructive pulmonary disease [12]. EPA could also diminish lipopolysaccharide and prostaglandin E(2)-stimulated TNF-α and IL-6 expression. Besides, EPA-treated macrophages repressed TNF-α and IL-6 production in hepatocytes. Consequently, EPA has both direct anti-inflammatory influence on macrophages and hepatocytes and indirect functions on inflammation process through modulation of activated macrophages [20].

On the other hand, former reports have shown the effects of TNF-α and IL-6 pro-inflammatory cytokines in epileptogenesis in animal model. Overexpression of these cytokines within astrocytes has led to decrease in seizure threshold as well as increase in spontaneous seizure frequency [21, 22]. In addition, TNF-α is among cytokines which participate in neuronal hyperexcitability through alterations in ion channels and glutamate production. TNF-α release by microglial cells following seizures triggers glutamate production from astrocytes, thus depolarizing their membrane potential [23]. Inflammatory response caused by IL-6 production in the central nervous system leads to spontaneous neurodegeneration and serious neurological damage following exposure to neurotoxins [24]. Consequently, decrease in the levels of these cytokines in patients supplemented with omega-3 FAs might contribute in anti-epileptic effects of omega-3 FAs. Such hypothesis is in line with the observation of reduction in seizure frequency following omega-3 FA supplementation in spite of no change in serum AED concentrations as revealed in Yuen et al. study [3]. However, Bromfield et al. have demonstrated no superiority for the administration of EPA plus DHA (2.2 mg/day in a 3:2 ratio) to placebo as complementary treatment for refractory epilepsy in a 12-week treatment period. Yet, they did not exclude the efficacy of different doses or different EPA:DHA ratios in this regard [25]. DeGiorgio et al. have also reported no significant change in seizure severity in patients with refractory seizure following administration of 9600 mg of fish oil/day (2800 mg of omega-3 FA) in their randomized, double-blind, cross-over clinical trial [26]. Considering the discrepancy between these studies, further studies with larger sample sizes are required to reach a consensus about the beneficial effects of omega-3 FAs in refractory seizure. We hypothesize that the duration of treatment might contribute in efficacy of omega-3 FA supplementation as Schlanger et al. have reported reduction in both frequency and strength of the epileptic seizures following 6 months duration of omega-3 FA supplementation [27].

More importantly, omega-3 FA supplementation in our study has resulted in improvement of EEG patterns in epileptic patients. Considering the predictive value of EEG patterns in some kinds of brain injury [16], the co-occurrence of improvement in clinical and paraclinical findings of epileptic patients supplemented with omega-3 FAs might be of practical significance. However, future studies are needed to assess the predictive value of EEG patterns retrieved at certain study points during the whole study period.

Despite the dominance of DHA in brain tissues compared with other FAs [5], omega-3 FA supplementation studies in patients with neuropsychiatric disorders have mostly demonstrated effectiveness of solitary administration of EPA [3]. As in the present study we assessed the effects of an available EPA and DHA preparation in refractory seizure, future studies are necessary to compare the efficacy of certain FAs in these patients. Our study has some limitations as we could not assess plasma levels of DHA and EPA in placebo- and Supplement-patients before and after treatment. We also state the small sample size as a limitation of our study which necessitates confirmation of the results in larger sample sizes.

## Conclusion

We demonstrated the beneficial effects of omega-3 FA supplementation in refractory seizures which were concomitant with reduction in serum levels of pro-inflammatory cytokines. Further studies are needed to evaluate the effects of different omega-3 FA products with focus on determination of the most effective dose and treatment duration.
